# Supplementary material for: Enhanced photon collection enables four dimensional fluorescence nanoscopy of living systems
Source: Nat Commun. 2018 Aug 16;9:3281. doi: 10.1038/s41467-018-05799-w (PMC6095837; doi:10.1038/s41467-018-05799-w)
Supplement: Supplementary file 1 — Supplementary Information [file 41467_2018_5799_MOESM1_ESM.pdf]

Supplementary Information for

Enhanced photon collection enables four dimensional fluorescence nanoscopy of living systems

Masullo et al.

correspondence to: ilaria.testa@scilifelab.se; testa@kth.se

Supplementary Notes 1 - Pinhole size comparison

In order to create a final image from any scanning microscope, the signal needs to be quantified in each step of the scan and assigned to the correct position in the final image. When using an image sensor as detector, a straight forward way to obtain a pinholed signal is to computational select the pixels from which the signal will be extracted and discarding the signal in any other pixel. Practically this can be done by element-wise multiplication between the array of pixel values and an array of values representing a digital pinhole followed by summation of all the value in the resulting array. This is analogous to a physically pinholed detection in a single point scanning microscope.

It is important to note that when using an image sensor or other array detector, each detector element (pixel) is associated to noise and summing more elements increases the total amount of noise i.e. by increasing the size of the digital pinhole, there is an inevitable increase in noise. An optimal pinhole size collects the maximum amount of signal while not accumulating unnecessary noise, giving a maximum signal to noise ratio.

With a sub-diffractive Gaussian emitting spot in the sample, the signal falling on the image sensor will have a Gaussian shape resulting from the convolution of the emitting Gaussian with the Gaussian PSF of the microscope. Considering a digital pinhole with a Gaussian shape, the total signal collected by the pinhole can be calculated as the integral of the product between the Gaussian signal and the Gaussian pinhole.

Contrary to the signal, the noise is not localized. Slightly simplified, we can consider the noise as identical independent random variables added to the true pixel values. The variance of the summed pixel values will scale linearly with the area of the pinhole and thus the standard deviation of the noise will scale linearly with the FHWM of the pinhole.

Taking the ratio between the signal and the noise gives a clear maximum SNR (see Figure 1F) when using a Gaussian pinhole with a FWHM of around 200 – 250 nm. The same conclusion can be drawn by seeing the signal falling on the sensor as a vector and the recorded pixel values as this signal vector with added noise. The best estimation of the scaling of the signal vector (the signal strength) in a least squares sense is then the projection of the recorded pixel vector onto the normalized signal vector (this then corresponds to the previously called pinhole) i.e. the scalar product between the two. The scalar product equates to element-wise multiplication followed by summation i.e. the same operation as the digital pinholing previously described.

Supplementary Notes 2 - Image reconstruction

MoNaLISA raw data consists of a stack of frames. Each frame contains the signal from an ensemble of spatially separated emitting foci (see Supplementary Figure 3). The reconstruction algorithm aims to quantify the amount of signal from the in-focus emitting foci in each frame and to assign this value to the pixels that compose the final super resolved image. The small areas of the camera sensor corresponding to individual foci and considered for signal quantification we call unit cells and the digital masking functions used in these unit cells we call digital pinholes.

In order to accurately quantify the emission from each emitter in the recorded frames, the illumination patterns need to be well characterized so that the digital pinholes can be centered precisely on the emitting points. This alignment procedure can be done manually before acquisition by precisely characterizing the location and periodicity of the microlens pattern on the image sensor. In order to optimize the workflow, we implemented an automatic alignment algorithm inspired by the work of Chmyrov et al. *(1)*. The algorithm uses the summed frames from the recording and matches a reference pattern to the pattern from the recording by maximizing the correlation between the two.

We now consider the object plane as the focal plane in the sample (see Supplementary Figure 3A) and the image plane as the plane on the camera sensor (see Supplementary Figure 3B). The image of each confined emitter in the object plane will be centered on the center of its corresponding unit cell and distributed spatially according to the PSF of the system, in our case approximated as a 2D Gaussian function.

The fact that the samples imaged are three dimensional means that apart from having photon emission from proteins in the focal plane, there can be a contribution from out of

focus planes. Hence, the signal in each unit cell of the raw frame, surrounding a signal center, consists of an in-focus Gaussian PSF plus a blurred, or even constant, background.

In the reconstruction software, we choose to model the signal distribution on each unit cell as a linear combination of two bases vectors representing an in-focus 2D Gaussian bell curve ($\mathbf{b_1}$) and a constant background ($\mathbf{b_2}$) (see Supplementary Figure 3E). The recorded data will then have added noise so that the recorded data $\mathbf{v}$ can be expressed as $\mathbf{v} = c_1\mathbf{b_1} + c_2\mathbf{b_2} + \mathbf{n}$, where $\mathbf{n}$ is a random noise vector and c_n are scaling factors. Given the acquired noisy data, the coefficients c_1 and c_2 that fit the model best in a least squares sense can be calculated as

$$c_1 = k_1\langle \mathbf{b_1}, \mathbf{v}\rangle + k_2\langle \mathbf{b_2}, \mathbf{v}\rangle \text{ and } c_2 = l_1\langle \mathbf{b_1}, \mathbf{v}\rangle + l_2\langle \mathbf{b_2}, \mathbf{v}\rangle$$

where k_1, k_2, l_1 and l_2 are the elements of the inverse Grammian matrix

$$\begin{pmatrix} k_1 & k_2 \\ l_1 & l_2 \end{pmatrix} = \begin{pmatrix} \langle \mathbf{b_1}, \mathbf{b_1}\rangle & \langle \mathbf{b_1}, \mathbf{b_2}\rangle \\ \langle \mathbf{b_2}, \mathbf{b_1}\rangle & \langle \mathbf{b_2}, \mathbf{b_2}\rangle \end{pmatrix}^{-1}$$

It is at this point interesting to note that the coefficient c_1 (which becomes our final image) is a linear combination of the scalar products between the data and different digital pinholes defined by $\mathbf{b_n}$. The scalar product between the data vector and a pinhole vector in the digital regime is equal to physically pinholing and quantifying the incoming light in a standard confocal microscope. Least squares fitting of the data to a model is thus equal to a differential detection scheme with different pinholes but with objectively optimized coefficients. Compared to physically pinholing in detection, the fact that our camera sensor acts as an array detector gives us additional information about the intensity distribution that we can use to improve the accuracy of our signal extraction. It is possible to further refine

the model, adding more basis functions (or digital pinholes) representing slightly out of focus emission that gives a slightly blurred PSF (*6*).

Supplementary Notes 3 - Bleaching and noise correction

During the acquisition of each frame, the rsFPs are cycled between their ON-OFF states causing them to fatigue and their mean photon emission rate to decrease. To compensate for this, bleaching correction is implemented in the reconstruction of the images. The correction is done after the extraction of the signal. The average signal decay over the time of the recording is calculated by summing the signal from all foci in each frame. The pixel value in each frame is scaled to compensate for this by dividing the pixel values with the relative averaged signal.

Owing to the negative switching of the rsFPs used in this MoNaLISA scheme, there is emission of light during the OFF-switching phase. This fluorescent light will reach the camera sensor before it is exposed and cause a specific type of noise from trapped charge in the pixels. The amount of trapped charge seems pixel dependent meaning that the different areas of the final image, detected on different pixels of the sensor, may show slight variations in intensity. We handle this issue by first identifying the average offset of each pixel during a scan caused by the inherent noise characteristics of that pixel. To do so we create an average image of all the frames collected during an acquisition. By using the built-in rolling ball background subtraction method in Image J with a ball radius of one pixel, a map of the hot pixels and their average offset can be created. This image can then be subtracted from each raw data frame to minimize the effect of this phenomenon. The same type of effect can also result from slightly varying intensities of the individual foci due to imperfections of the microlens arrays. To further correct for this, we developed a customized correction algorithm based on averaged intensity matching at the edges of each

sub-square of the final reconstruction. The combination of the described corrections successfully suppresses the effect and gives a homogeneous reconstruction.

Once the signal has been extracted from each raw frame in the scanning procedure, signal values are directly translated into pixel values and assigned to its correct position in the final image. Some small adjustments can also be made in the pixel assignment process to correct for slight imperfections in the physical motion of the scan.

Supplementary Notes 4 - Enhanced optical sectioning

The optical sectioning capability of MoNaLISA imaging set-up comes from the use of modulated and decoupled light patterns in all three steps of illumination, which confines the emission in all three spatial dimensions. The modulated ON-switching and read-out illumination leads to a confined emission in both the focal plane (further confined by the OFF-switching pattern) and along the optical axis (see Supplementary Figure 4A).

Considering the case where the states are far from saturation, both ON-switching and excitation are linear processes but the total fluorescent signal is of quadratic order, proportional to the combined effect of both 405 nm and 488 nm illumination. Thus, the effective emitting volume can be estimated as the product of the ON-switching PSF and the excitation PSF. For the axial dimension we have:

$$Exp\left(-\frac{(z-z_0)^2}{2\sigma_{\mathrm{eff}}{}^2}\right) \approx Exp\left(-\frac{(z-z_0)^2}{2\sigma_{405}{}^2}\right) \times Exp\left(-\frac{(z-z_0)^2}{2\sigma_{488}{}^2}\right)$$

$$\sigma_{\mathrm{eff}} = \sqrt{\frac{\sigma_{405}{}^2 \times \sigma_{488}{}^2}{\sigma_{405}{}^2 + \sigma_{488}{}^2}}$$

Where $\sigma = \frac{\mathrm{FWHM}}{2.355}$ and FWHMz $\approx \; 2\lambda/\mathrm{NA}^2$. Thus, for an NA=1.4 we will have $\sigma_{405} =$ 175 nm, $\sigma_{488} = 211$ nm and $\sigma_{\mathrm{eff}} = 136$ nm.

This yields

$$\mathrm{FWHMz}_{\mathrm{eff}} = 2.355\,\sigma_{\mathrm{eff}} \approx 320\ \mathrm{nm}$$

According to this estimation, the minimal axial confinement is ~ 320 nm

Since in MoNaLISA imaging we have simultaneous emission from multiple foci, the optical sectioning ability will not only depend on their individual spatial confinement but also on their separation. To quantify MoNaLISA true sectioning ability and to compare it

with existing methods, we developed a model. Here, we consider multiple pointlike emitters in the x-y plane with a certain elongation along the optical axis.

The signal from each emitting spot is detected and measured at its corresponding coordinates on the image sensor. But since there is simultaneous emission from all emitters and potentially also from different depths of the sample, each digital pinhole may detect light emitted from different planes and different emitting spots. The part of the light falling on a digital pinhole that originates from its corresponding in-focus emitter we call the in-focus signal. The in-focus-signal is distributed on the image sensor as a diffraction limited Gaussian PSF (see Supplementary Figure 4C) and its intensity is centered on the digital pinhole. The part of the signal falling on a digital pinhole that is not the in-focus signal is defined as the out-of-focus signal. Pointlike emitters in an out-of-focus plane will be imaged according to a different PSF determined by the plane's *z*-position. We choose to model the out-of-focus PSF as a Gaussian function with increasing area (but constant total intensity) as the distance from the focal plane increases (see Supplementary Figure 4D). In the following model, we investigate the signal contribution from individual out-of-focus planes, depending on the distance between individual emitting spots in the focal plane and along the optical axis.

In order to quantify the difference between a uniform (WF-RESOLFT, see Supplementary Figure 4B) and a confined (MoNaLISA, see Supplementary Figure 4A) ON-switching and read-out, we consider two separate planes in the sample, one at $z = 0$ i.e. in focus, and one at a certain distance z out of focus. In each of the planes, we consider the emission from pointlike sources. The ensemble of point emitters in the plane will result in a sum of shifted PSFs on the image sensor. The center of a digital pinhole on the camera

sensor will thus in this situation see not only light emitted from its corresponding in-focus emitting point, but also from one or more emitting points residing in the out-of-focus plane. The ratio between the desired in-focus signal and the total signal, including the unwanted out-of-focus signal is what we choose to use as a measure of the contrast between the in-focus and the out-of-focus planes. We then calculate:

$$C(z,d) = \frac{S_{\text{i.f.}}}{S_{\text{i.f.}} + S_{\text{o.o.f}}(z,d)}$$

$$S_{i.f.} = \text{Em}(0) * \text{PSF}(0,0,0)$$

$$S_{\text{o.o.f}}(z,d) = \sum_{n=-\infty}^{\infty} \sum_{m=-\infty}^{\infty} \text{Em}(z) * \text{PSF}(n*d, m*d, z)$$

where $C(z, d)$ is the contrast between an in-focus plane and an out-of-focus plane which is at a distance z from the focal plane and d is the distance between emitting spots in both lateral dimensions x and y (we considered the case of a squared multi-foci pattern). $S_{\text{i.f.}}$ is the in-focus signal intensity, $S_{\text{o.o.f.}}$ is the out-of-focus signal intensity, Em(z) is the relative emittance from an emitter at position z and PSF(x, y, z) is the approximated three-dimensional detection PSF of a point emitter. For WF-RESOLFT, Em(z) = 1, meaning homogenous uniform activation and excitation.

We fix the period d as d = 750 nm for MoNaLISA and d = 250 nm for WF-RESOLFT such as what would arise from using an OFF pattern with P_{SW} = 250 nm in both cases and P_{MF} = 750 nm for MoNaLISA. Using these parameters, the value of *C(z)* can be calculated

for both MoNaLISA and WF-RESOLFT (see Supplementary Figure 5A). The plot of a theoretical single point scanning setup, which corresponds to setting $d \to \infty$ in the MoNaLISA case, is also shown for comparison.

From these calculations we can conclude that the contrast between the signal from in-focus structures and out-of-focus structures increases significantly faster in the MoNaLISA case than in the WF-RESOFLT as the distance between the planes increases. MoNaLISA performs equivalently to a point-scanning setup (PS-RESOLFT) while WF-RESOLFT never reaches a contrast above ~ 0.54. Another way to interpret the graph is to quantify the area between Contrast = 1 and the curves. This area is a relative measure of the total potential background emitted from all the out-of-focus planes, assuming a sample with high label density in all planes. For MoNaLISA/PS-RESOLFT, the total background is bound upwards and independent of the sample thickness, whereas the background in WF-RESOLFT increases linearly with sample thickness causing low contrast imaging.

Supplementary Notes 5 - Multi-foci P_{MF} optimal periodicity and 3D distribution

We have until now considered a MoNaLISA imaging scheme with a fixed P_{MF} = 750 nm. In this section we discuss the impact of the parameter P_{MF} on the systems optical sectioning ability. We first consider the same model as described above, but instead we plot the contrast as a function of P_{MF} for three different *z*-planes.

The graph in Supplementary Figure 5B suggests that for all the *z*-planes plotted, the contrast does not increase significantly when increasing the distance above ~ 500 nm. The reason we chose P_{MF} = 750 nm is due to the 3D distribution of the multi-foci light patterns.

So far we have considered the multi-foci pattern to be an ensemble of independent foci. This model holds very well for the focal plane and its immediate vicinity, but for planes that reside several wavelengths away from the focal plane, the 3D extensions of the different PSF's will start to overlap. Since all foci are created from coherent light they will interfere and can create highly modulated patterns away from the focal plane as well. Experimental measurements show that for the magnification used to create a multi-foci periodicity of 750 nm in the focal plane there is a pattern repetition along the optical axis every 1.2 µm (see Supplementary Figure 6A–C). Importantly, in every second repetition, there is a phase shift relative to the in-focus pattern by half a period. Supplementary Figure 6D–F shows how the patterns are likely to look on a larger field of view. The same characteristics are observed for illuminations at 405 nm and 488 nm. The intensities of the pattern at different planes decreases as the distance from the focal plane increases. We interpret this behavior to be caused by a lower detection efficiency at planes away from the focal plane and bleaching of the fluorescent beads.

To investigate the influence of these potential out of focus emitter on the image quality we consider the interplay between these 3D patterns, including the OFF-pattern. The null volumes of the OFF-pattern are located every 250 nm in *x*-*y* and elongated along *z* (where the *z* dimension is parallel to the optical axis). If the maxima of the foci are coaligned with the nulls of the OFF-pattern in the focal plane, then the intensity maxima in the first repetition of the focal plane (at z = 1.2 μm) will be coaligned with the maxima of the OFF-pattern. The first repeated plane will thus be switched off and will not contribute to any significant background.

Moving to the second repetition plane, located at z = 2.4 μm, the foci in this plane will again be coaligned with the null of the OFF-pattern meaning emittance from these point is not silenced. If there are structures at the locations of the repeated planes, these will give rise to a minor background contribution. This contribution, though, is likely to be small enough not to affect the optical sectioning ability of the microscope.

If, however, we choose a smaller periodicity *d* of the foci pattern in the focal plane, it will result in a shorter repetition rate along the optical axis. Since the magnification along the optical axis scales quadratically with the magnification in *x*-*y*, only decreasing the periodicity in the focal plane to 500 nm instead of 750 nm (a demagnification factor of 1.5) would bring the first repetition in *z* to 533 nm instead of 1200 nm (a demagnification factor of 1.5^2). On top of this, staying with an OFF-switching periodicity of 250 nm would mean that the first repetition along *z* would no longer coincide with maxima of the OFF-pattern but with the minima. This would cause a large increase in background leading to worse image quality. Supplementary Figure 7 shows the distinct increase in total background as

a larger area between the curve and the $C = 1$ line, increasing further as the sample thickness increases.

The better optical sectioning achieved with $d = 750$ nm compare to $d = 500$ nm is further demonstrated in the simulation presented in the following section and by the experimental data recorded at different P_{MF} shown in Supplementary Figure 8.

Supplementary Notes 6 - Simulations at different multi-foci periodicity

To further consolidate our theory, we created a simulation tool to simulate data acquired from sequential switching and detection of a fluorescently labeled three-dimensional sample.

The simulated sample is made from a 10 x 10 x 4 μm^3 volume of voxels where the voxel value represents the relative amount of rsFPs in that position. To simulate a structure, we create a geometric pattern of ones as shown in Supplementary Figure 9A–B. This structure is composed of straight lines (filaments) with varying separation (~80–300 nm) in planes separated by 300 nm along the optical axis. The central plane is placed in the plane representing the focal plane. Each plane is rotated ten degrees with respect to the previous. The volume between the planes that contain the structure is filled with the value 0.01 to represent fluorescence from potential cytosolic rsFPs and autofluorescence. The light patterns are created as three-dimensional relative intensity distributions over a volume of equal size to the sample. The simulated sample and light patterns are created with a voxel size of 20 x 20 x 20 nm^3.

To describe the switching of the proteins, we consider the total energy delivered to a specific coordinate as the product between a relative energy of the illumination pulses (E_{ON}, E_{OFF}, and E_R) and the relative intensity distributions of the patterns ($I_{ON}(x,y,z)$, $I_{OFF}(x,y,z)$, and $I_R(x,y,z)$). Note that in practice, the E-values can be tuned by either changing the pulse duration or the output power of the laser. The activation of the rsFPs is then described mathematically as

$$\mathrm{ON}_{\mathrm{pre}}(x,y,x) = S(x,y,z) * (1 - Exp\big(-E_{\mathrm{ON}} * I_{\mathrm{ON}}(x,y,z)\big))$$

Where $\mathrm{ON_{pre}}(x,y,z)$ is the distribution of ON-state rsFPs after illumination with ON-switching light of the distribution $I_{\mathrm{ON}}(x,y,z)$ with energy E_{ON}, and $S(x,y,z)$ is the normalized sample label density. The distribution of rsFPs still in the ON-state after illumination with OFF-switching light ($\mathrm{ON_{post}}$) is then

$$\mathrm{ON_{post}}(x,y,z) = \mathrm{ON_{pre}}(x,y,z) * (\mathrm{bg} + (1-\mathrm{bg}) * Exp\big(-E_{\mathrm{OFF}} * I_{\mathrm{OFF}}(x,y,z)\big))$$

Where bg is the background level to which the OFF-switching converges as the energy delivered increases (residual ON-state proteins). $I_{\mathrm{OFF}}(x,y,z)$ is the intensity distribution of the OFF-switching pattern and E_{OFF} is the energy of the OFF-switching pulse. The emission from illumination with the read-out pattern thus becomes

$$\mathrm{Em}(x,y,z) = \mathrm{ON_{post}}(x,y,z) * \int_0^{E_R} \Big(\mathrm{bg} + (1-\mathrm{bg}) * Exp\big(-E_{\mathrm{R}} * I_{\mathrm{R}}(x,y,z)\big)\Big)\, dE_{\mathrm{R}} =$$

$$\mathrm{ON_{post}}(x,y,z) * (-Exp\big(-E_{\mathrm{R}} * I_{\mathrm{R}}(x,y,z)\big) + \mathrm{bg} * Exp\big(-E_{\mathrm{R}} * I_{\mathrm{R}}(x,y,z)\big) + \mathrm{bg} * E_{\mathrm{R}} - b + 1)$$

Where E_{R} is the energy of the read-out pulse and $I_{\mathrm{R}}(x,y,z)$ is the read-out pattern intensity distribution. Note here that since E_{R} is the product of time and intensity, integrating over E_{R} is analogous to integrating over time if the intensity if constant.

The transformation from the three-dimensional emission function to an intensity distribution on the camera sensor is simulated by summing up the emission from each emitting plane after the emission is blurred with a Gaussian filter of varying size depending on the *z*-position of the emitting plane. Between each step in a simulated scan, the

underlying sample, or label density distribution, is moved in accordance with the simulated scan. We added poissonian noise to the measurements to generate the final simulated data. By scaling the detected signal before adding the poissonian noise we simulate different emission brightness of the rsFPs. In these simulations, we assume ideal detection meaning we do not simulate any noise originating from the detector (in our case the camera) such as read-out noise or dark noise.

We simulate data using both wide-field ON-switching and read-out (WF-RESOLFT) and with the multi-foci pattern used in MoNaLISA. The energy used in the widefield switching corresponds to the peak energy of the multi-foci patterns for both ON-switching and read-out. For MoNaLISA and WF-RESOLFT simulations, we use an OFF-pattern with a periodicity of 250 nm. The peak intensity of the two OFF-switching patterns are the same in both cases. In the simulations, the multi-foci patterns are modelled as the sum of individual foci repeated at distance d in the focal plane and also repeated along the optical axis according to the model described in the previous section.

Simulated data was created for different brightness of the emitters, giving different signal to noise ratios in the reconstructed images. The superior in-focus signal detection of the MoNaLISA approach with P_{MF} = 750 nm can be clearly seen from the results of the simulations (see Supplementary Figure 9C–H). In the top MoNaLISA image (see Figure 9C), the signal is just strong enough to enable separation of the most closely packed lines in the MoNaLISA image, whereas in the WF-RESOLFT image (see Figure 9E), the in-focus signal is so low that even the well separated filaments are drowned out by the noise from the background emission. When drastically increasing the brightness of the emitters by a factor of 30, the tightly packed filaments become resolvable even from the

WF-RESOLFT data (see Supplementary Figure 9H). The image quality though at this brightness is still far superior in the MoNaLISA image (see Supplementary Figure 9F).

The simulation tool also helps to further explore the increased impact of the repeated planes along the optical axis when moving from P_{MF} = 50 nm to P_{MF} = 500 nm. For both the levels of brightness considered, it is clear that MoNaLISA with 500 nm periodicity (see Supplementary Figure 9D and Supplementary Figure 9G) show inferior image quality compared to MoNaLISA with 750 nm periodicity (see Supplementary Figure 9C and Supplementary Figure 9F).

Supplementary Notes 7 - Cell viability measurements

We tested potential light induced phototoxic effects during live cell MoNaLISA imaging with three different approaches. Firstly, we applied a LIVE/DEAD assay just after MoNaLISA imaging to check for cell viability. Secondly, we carefully checked the mitochondria networks morphology over the entire cell before and after MoNaLISA imaging to carefully seek for any sign of light induced stress. Finally, we looked at the cell edges to see if light induced shrinking and retractions happened. All the experiments have been performed on multiple cells. We imaged the human knock-in cell line expressing the rsEGFP2 fluorescently tagged vimentin.

In the first experiment, we used the LIVE/DEAD viability/cytotoxicity kit (Thermo Fisher Scientific, L3224). This assay allows the discrimination between live and dead cells, based on the detection of cytosolic (green live) or nuclear (red dead) fluorescence signal localization. Calcein-AM dye emits green-fluorescence by detecting the intracellular esterase activity in live cells, while ethidium homodimer dye emits red-fluorescence by binding to the DNA in the cell nucleus of those cells with a compromised plasma membrane, allowing the detection of dead cells (2, 3). The cells were grown on 18mm cover glasses for 24h at 37 °C in 5% of CO2 and then imaged with MoNaLISA for 30 consecutive frames (see Supplementary Figure 11). The MoNaLISA power exploited were comparable with the typical setting used for a time lapse recording of rsEGFP2 (Table S1): ON-switch for 0.5 ms at 0.35 kW/cm^2, OFF-switch for 1.5 ms at 0.32 kW/cm^2 and excitation for 1 ms at 1 kW/cm^2 for a scan step size of 40 nm. Shortly after imaging, cells were stained with LIVE/DEAD solutions, following manufactures guidelines and were incubated for 30 min at 37 °C. Subsequently, wide-field images in both the red (620/60

nm) and the green (535/70 nm) channels were taken on the very same cell. A total number of N=15 cells, taken from different samples and measured over different days, has been tested; all of the cells showed a clearly green fluorescent cytosolic staining, and no red nuclear staining (see Supplementary Figure 11C). The dyes were previously validated on controls samples. The positive control, where the staining solution was added to the cells directly after 24h of culturing, with no previous imaging on them, showed a green signal similar to the one observed in the previous experiments and no red fluorescence was detected (see Supplementary Figure 11A). Two different negative controls have been analyzed. In the first one, all of the cells were treated for 10 minutes with Methanol resulting in a homogenous red staining of all of the cell nuclei and no green fluorescence was detected (see Supplementary Figure 11B). In the second one, one cell was chosen and was illuminated with a 10 times higher light dose then in MoNaLISA (see Supplementary Figure 12E), followed by the LIVE/DEAD staining. The red-fluorescence in the cell nucleus indicates the death of the cell.

In the second experiment, potential changes in the mitochondrial morphology were monitored during MoNaLISA imaging acquisition. It is known, indeed, that mitochondria morphological changes can be associated with the initial steps of the apoptotic process; in particular, an increase rate of fission and the formation of punctiform and fragmented mitochondria are indicators of cell stress (4, 5). We labeled the mitochondria with the MitoTracker CMXRos (Thermo Fischer Scientific, M7512). This fluorescent dye stains mitochondria in live cells and its accumulation is dependent upon active membrane potential. The knock-in cells were grown for 24h at 37 °C in 5% of CO2 and then incubated for 10 minutes with 10nM of MitoTracker CMXRos for mitochondria staining. An image

of the mitochondria (red channel 620/60 nm) was taken before and after recording 30 consecutive frames of the fluorescent vimentin (green channel 535/70 nm), using the imaging scheme of MoNaLISA. We imaged N=13 cells in total and among them 11 cells did not show any significant changes in the morphology of mitochondria after the MoNaLISA prolonged illumination (see Supplementary Figure 12A). The same experiment, conducted in a confocal mode without OFF-switching intensity, produced similar results demonstrating that the OFF intensities needed to improve the spatial resolution do not play a key role in possible phototoxic effects (see Supplementary Figure 12B). As negative control, we applied an illumination light dose 10 times higher than the one used in previous long MoNaLISA time lapse that caused a visible mitochondria fragmentation and cell death (see Supplementary Figure 12C).

Finally, we looked at the cell cytoskeleton by labeling LifeAct, an actin binding protein, and vimentin. We checked for the cell shape and borders during the typical MoNaLISA imaging intensities again, prolonged for 30 frames. By comparing the first and last frames we could observe movements but not overall retraction and shrinking of the cells, which indicate that the light doses during MoNaLISA imaging do not have an immediate light induced phototoxic effect. Also, a previous study on phototoxic effect by super resolution microscopy has highlighted that rsEGFP2, among other rsFPs, enables photodamage-free super resolution imaging over longer time periods, thanks to the lower UV light required for ON-switching (6).

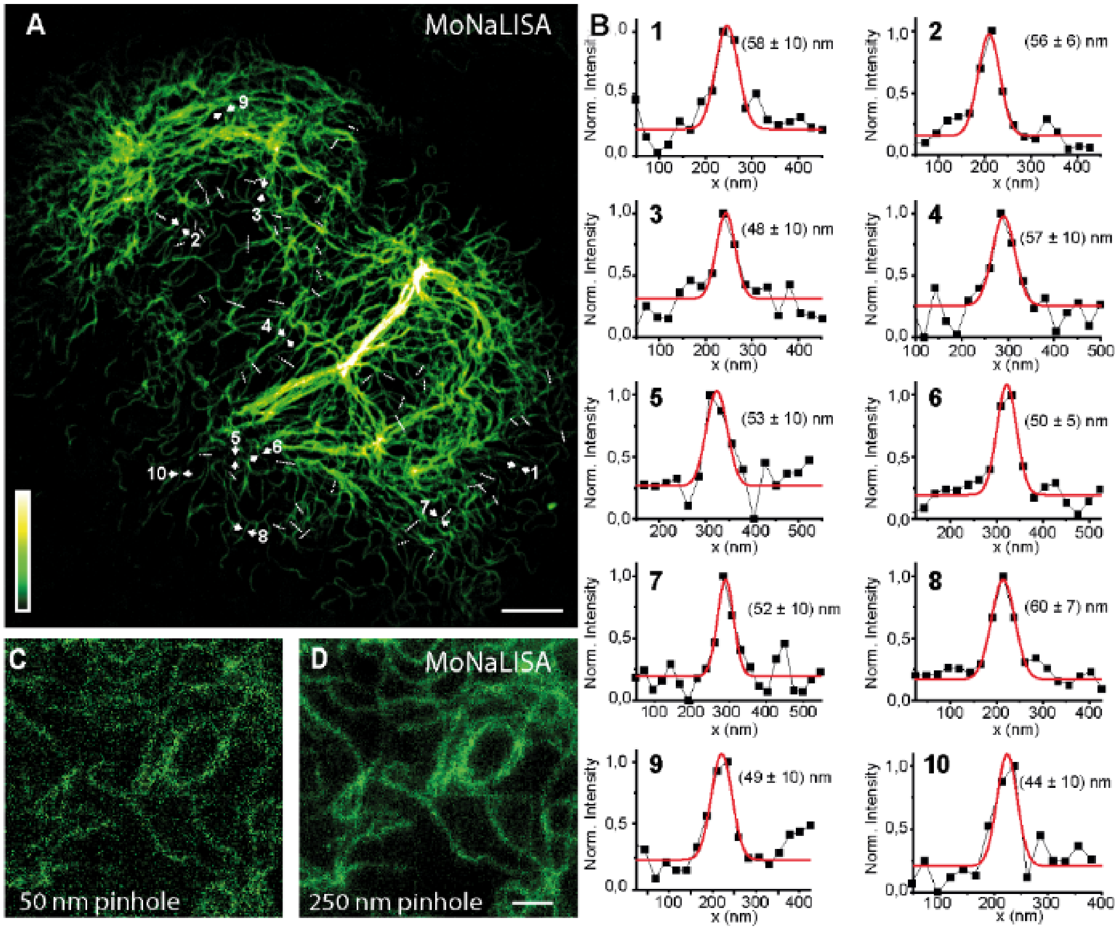

Supplementary Figure 1. Spatial resolution and image contrast. (**A**) MoNaLISA image of living U2OS cells endogenously expressing vimentin-rsEGFP2 reported in Figure 2 of the main text. Scale bar, 5 µm. In the image all the measured line profiles are shown (each averaged of 4 adjacent pixel lines corresponding to a filaments length of 100 nm). The resulting FWHM values were used to generate the histogram in Figure 2E. (**B**) Measured data fitted with Gaussian curves and related FWHM are reported for 10 representative lines profiles that are marked in the image (**A**) (FWHM ± s.e.m). (**C**) Image generated with a 50 nm digital pinhole, as commonly used in WF-RESOLFT to minimize crosstalk, compared to (**D**) image reconstructed with a Gaussian digital pinhole with a FWHM of 250 nm used in MoNaLISA. Scale bar, 1 µm.

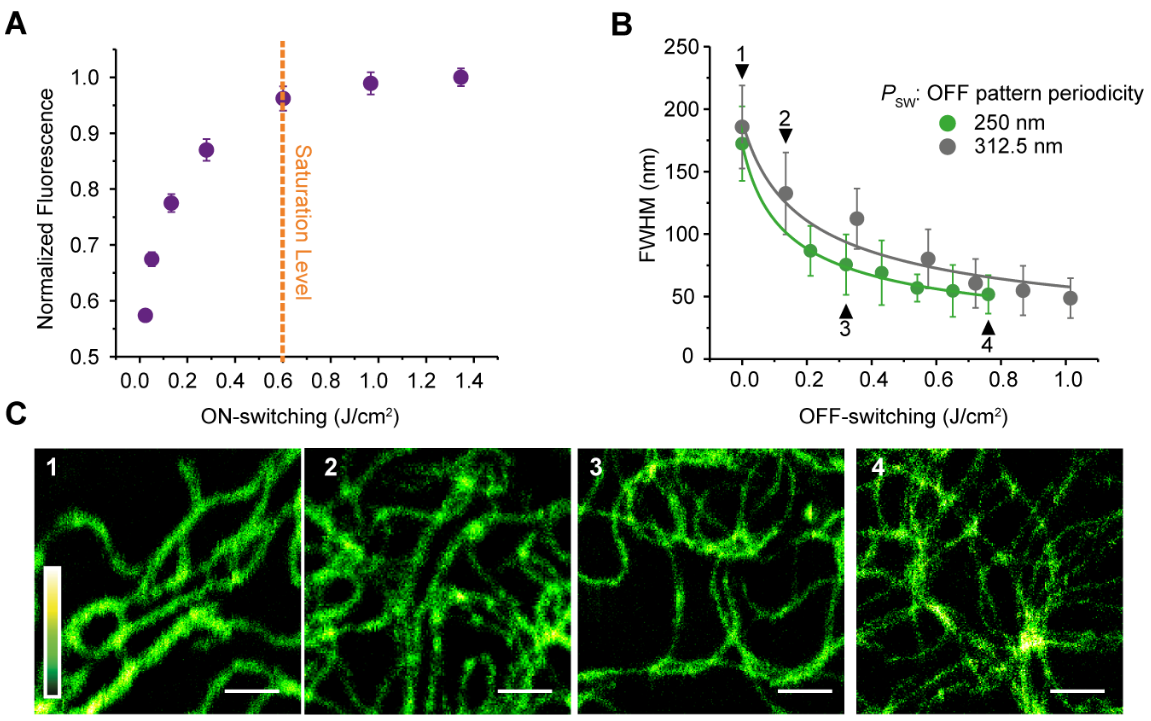

Supplementary Figure 2. Switching kinetics and spatial resolution. (**A**) ON-switching kinetics as a function of the 405 nm light doses. The experiments are performed in U2OS cell endogenously expressing vimentin-rsEGFP2. Each data point is the average of 10 cycles of the protein. Error bars indicate ± s.d. In each cycle the rsFPs were illuminated with 488 nm OFF-light for 50 ms at 170 W/cm^2 after initial ON-switching with 405 nm light for 1 ms at different powers. (**B**) Dependence of the spatial resolution on the OFF-switching light doses for two different OFF pattern periodicities (P_{SW}). The OFF-pattern with the smaller P_{SW} reaches the same resolution with lower doses of light. Each data point results from the Lorentzian fit of 25–30 filaments per OFF illumination. Error bars indicate ± s.d. The continuous curves are fitted depletion curves on the form 1/√(1+b). Each profile is the averaged of adjacent pixel lines corresponding to a filament

length of 80–140 nm. (**C**) Four representative images recorded with different OFF illuminations. Scale bar, 1 μm.

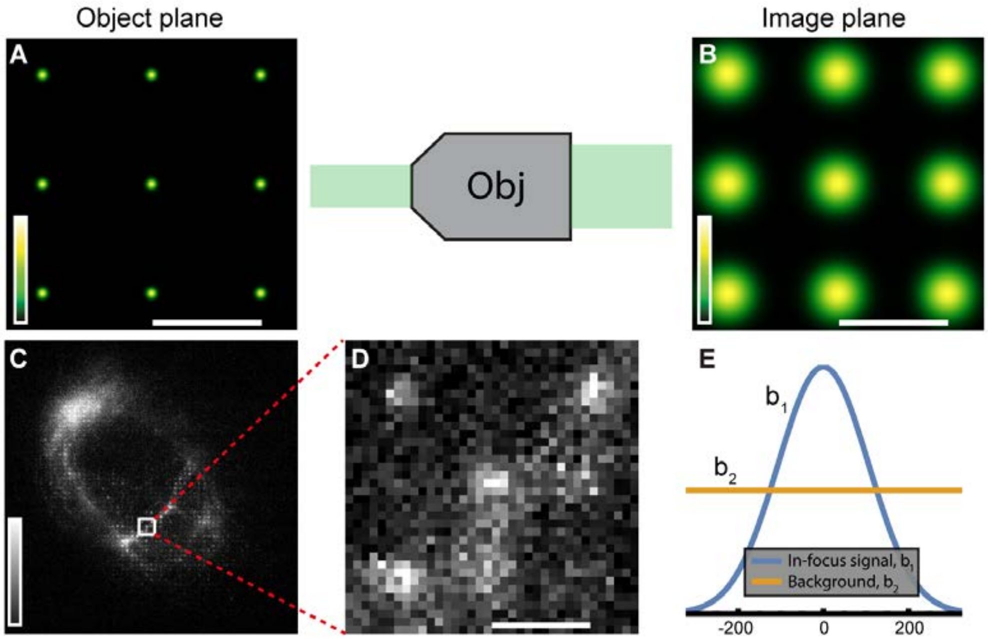

Supplementary Figure 3. Image reconstruction (**A**) The emission from the object plane is confined to sub-diffractive regions. (**B**) When imaged onto the image plane, each emitter is blurred according to the emission PSF of the microscope and centered on its corresponding digital mask. Images (**C**) and (**D**) show a raw frame from a recording. To estimate the signal emitted from the in-focus emitters, the ensemble of pixel values within each sub-area is modelled as a constant background plus an in-focus Gaussian PSF (**E**). This model consisting of the bases $\mathbf{b_1}$ and $\mathbf{b_2}$ is then fitted to the data in a least squares sense to acquire the estimated in-focus signal. All scale bars, 750 nm.

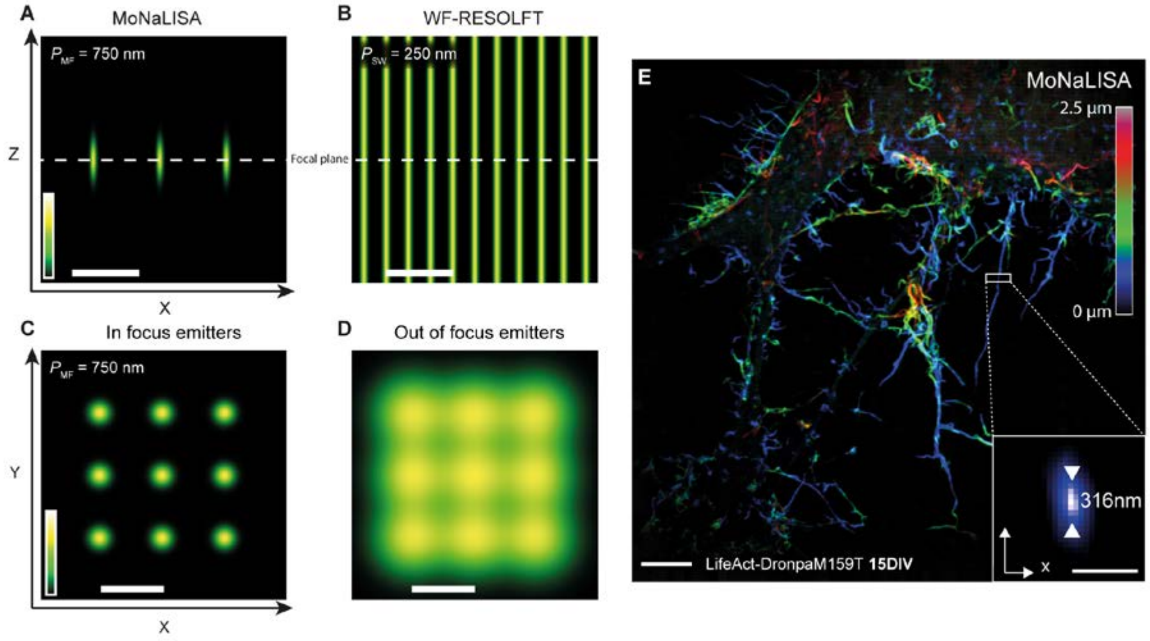

Supplementary Figure 4. **MoNaLISA and WF-RESOLFT spatial confinement of the fluorescence emission.** Calculated spatial distribution of the emitting volumes for MoNaLISA (**A**) and WF-RESOLFT (**B**) showing a clear signal confinement along the optical axis or MoNaLISA. Calculated images of point-emitters in the focal plane (**C**) and of out-of-focus emission (**D**) corresponding to a larger PSF. Scale bars, 750 nm. (**E**) 3D stacks of 15 DIV hippocampal neuron expressing LifeAct-DronpaM159T with MoNaLISA. The fine optical sectioning reveals the color-coded depth information. The inset is the x-z maximum projection of an actin filament. The Gaussian fit of the filament axial extension results in a FWHM of 316 nm, a factor 1.4 better than conventional sectioning. Scale bars, 5 µm (large image), 500 nm (inset).

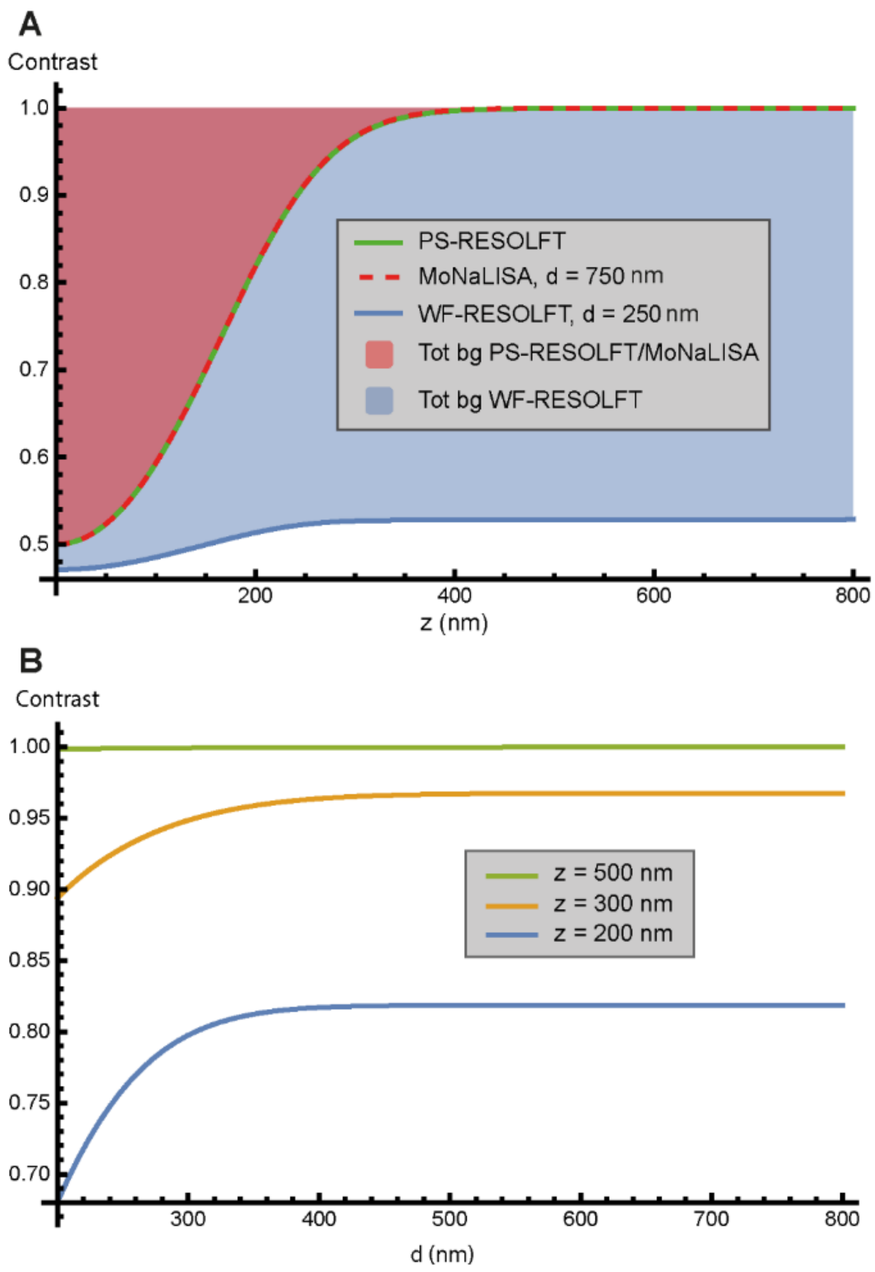

Supplementary Figure 5. Calculation of the in focus and out-of-focus contribution for different imaging modes. (**A**) Contrast *C* as a function of the distance z from the out of focus plane to the focal plane for PS-RESOLFT (point., WF-RESOLFT and MoNaLISA imaging. (**B**) Contrast *C* as a function of the distance d between emitting spots. The three curves refer to three different z-positions of the out-of-focus plane.

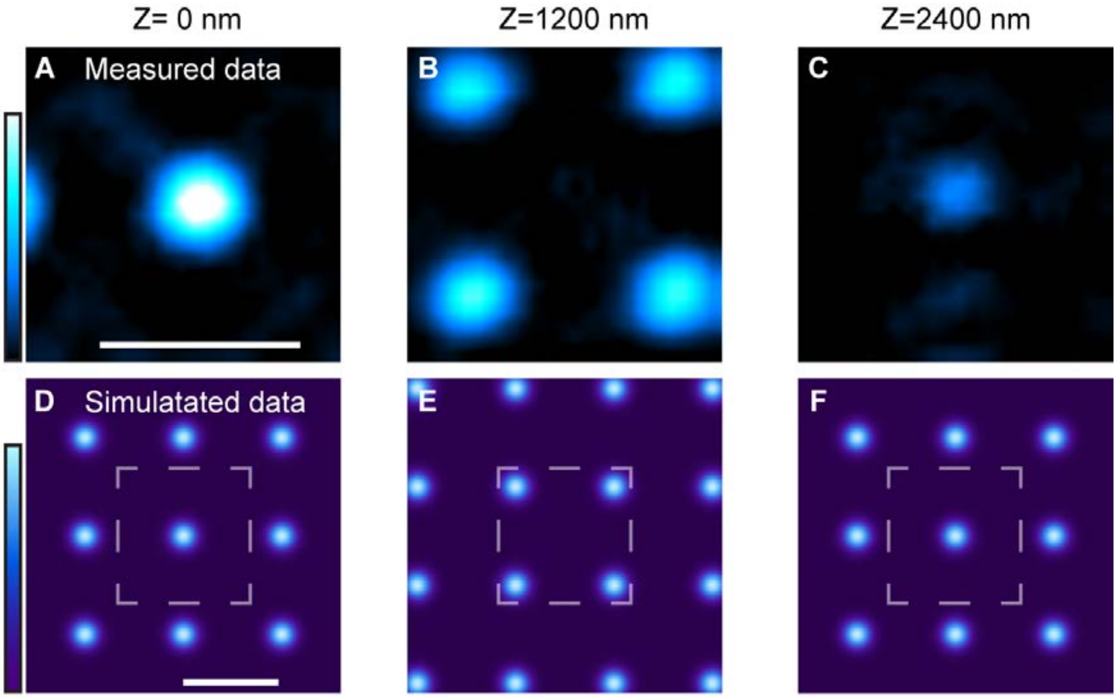

Supplementary Figure 6. ON-switching and read-out interference patterns repetition along the optical axis. By scanning a fluorescent bead over a volume we reconstructed the x-y intensity distribution of the multi-foci light pattern in 3D. We measured a multi-foci distribution at 0 nm (**A**), 1200 nm (**B**) and 2400 nm (**C**) position along the optical axis z. (**D–F**) simulations of the multi-foci interference patterns in the same planes. Scale bars, 750 nm.

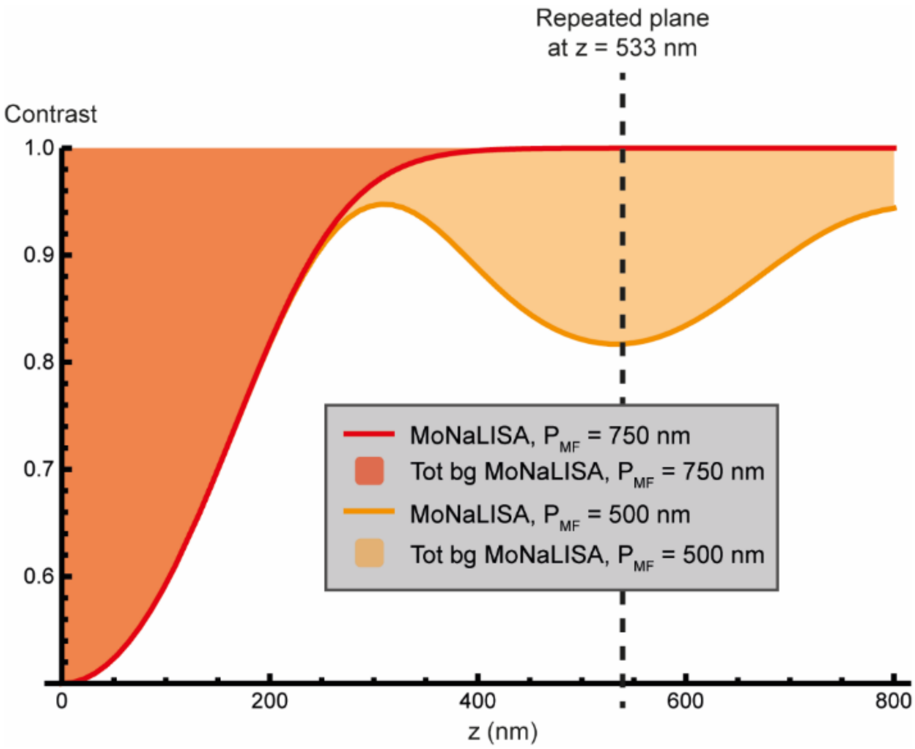

Supplementary Figure 7. Theoretical contrast with 3D pattern. The contrast *C* as a function of the distance z from the focal to the out-of-focus plane including in the model the repeated plane along the optical axis. P_{MF} is set to 500 nm and 750 nm respectively. The increased in periodicity in x-y of a factor 1.5 leads to a square factor 2.25 along the optical axis. Therefore, the first repeated plane is found at z = 533 nm with P_{MF} = 500 instead of 1200 nm with P_{MF} = 750 nm.

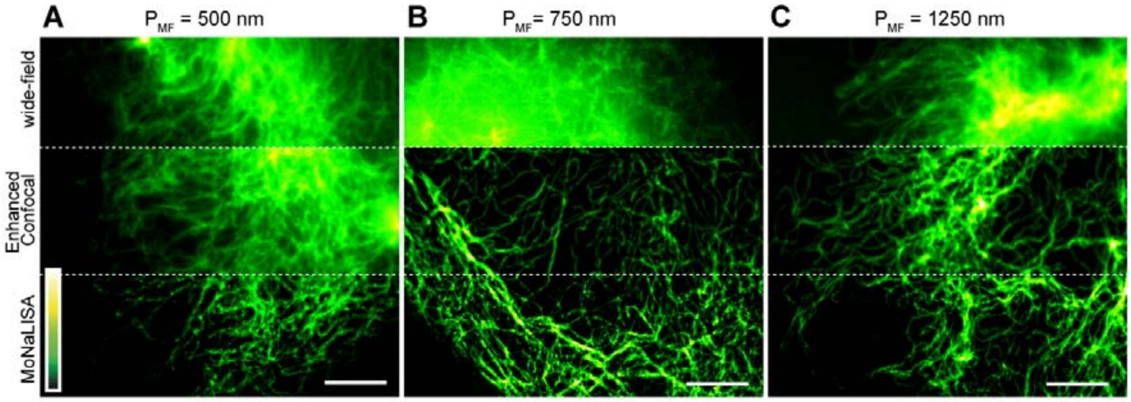

Supplementary Figure 8. MoNaLISA imaging at different multi-foci periodicity P_{MF}. All the images show U2OS cells endogenously expressing vimentin-rsEGFP2. The three cells in A, B, C were imaged with different P_{MF}: 500 nm (**A**), 750 nm (**B**) and 1250 nm (**C**) and the same OFF switching pattern with P_{SW} = 250 nm. Also, each cell was recorded with different imaging modalities: wide-field (up section), Enhanced Confocal (middle section) and MoNaLISA (bottom section). At P_{MF} = 500 nm the out-of-focus background is still visible. For P_{MF} > 750 nm we observed a clear optical sectioning effect, which is kept by further increasing the periodicity. Those experimental results support the theoretical calculation presented in section 5 of the supplementary notes. Scale bars, 5 μm.

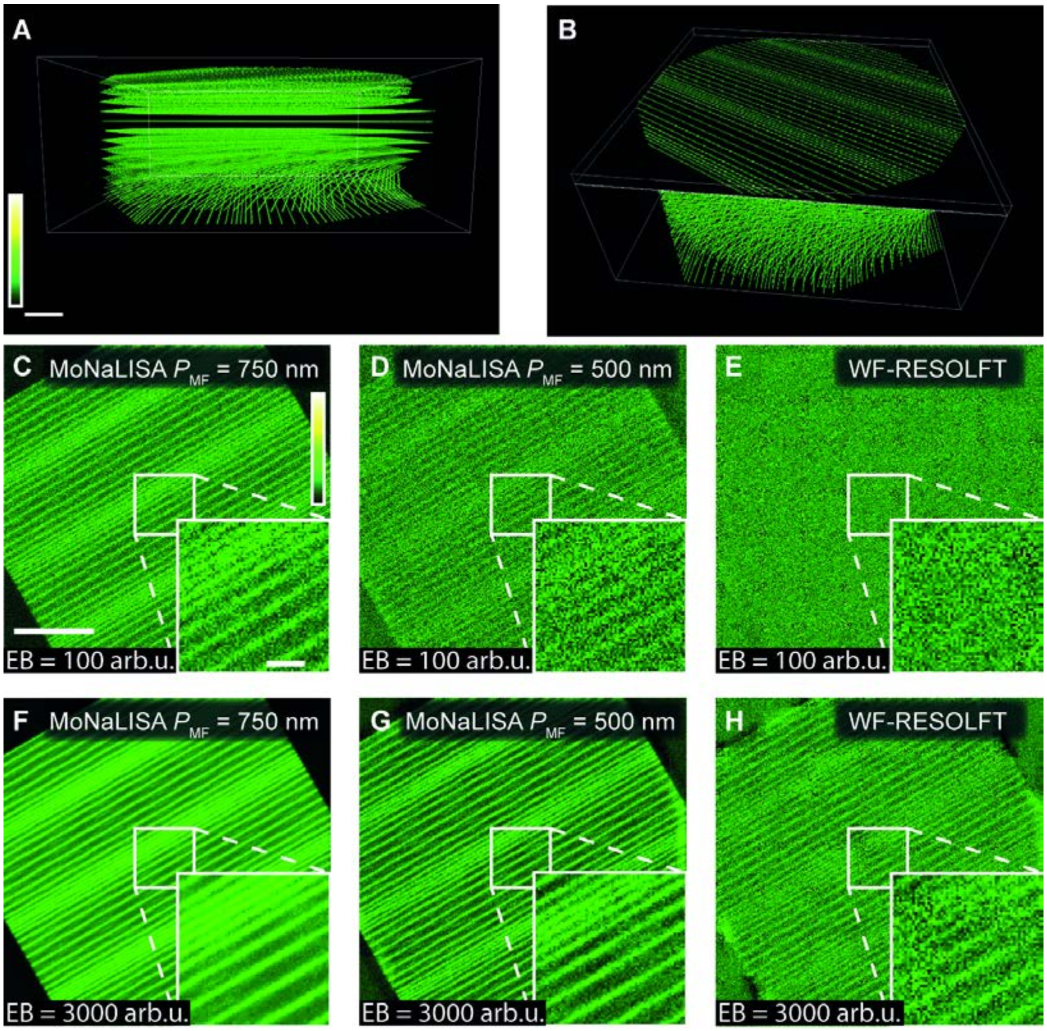

Supplementary Figure 9. Simulations (**A–B**) Two different views of the mock sample featuring multiple planes 300 nm apart along the z-axis. Each plane consists of straight parallel lines with varying separation. Scale bar approx. 1.5 µm. **(C-E)** Simulated imaging using MoNaLISA with 750 nm periodicity, MoNaLISA with 500 nm periodicity and WF-RESOLFT at emitter brightness of 100 arbitrary units. Scala bars in **(C)**, 2.5 µm (large image) and 500 nm (inset). **(F-G)** Simulated imaging using MoNaLISA with 750 nm periodicity, MoNaLISA with 500 nm periodicity and WF-RESOLFT at emitter brightness of 3000 arbitrary units.

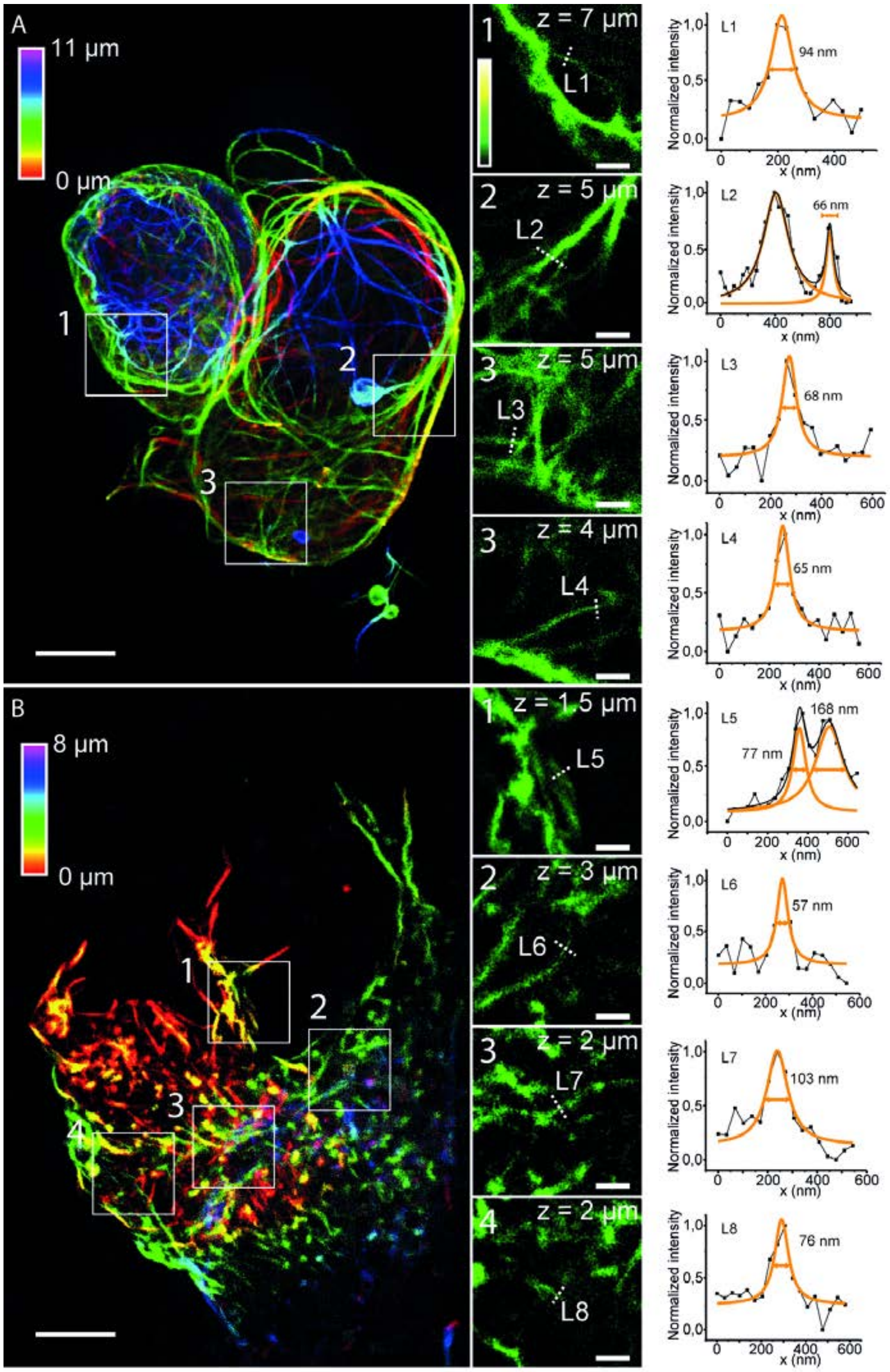

Supplementary Figure 10. Measurement of small actin bundles in 3D cells. Stem cell (**A**) and Astrocyte (**B**) with color coded depth information showing three and four magnified regions (right). Each magnified region is a single plane at marked z-position.

Eight representative line profiles have been drawn and the corresponding data were plotted and fitted with Lorentzian curves in the adjacent graph. Scale bars, 5 μm (large images) and 1 μm (small images).

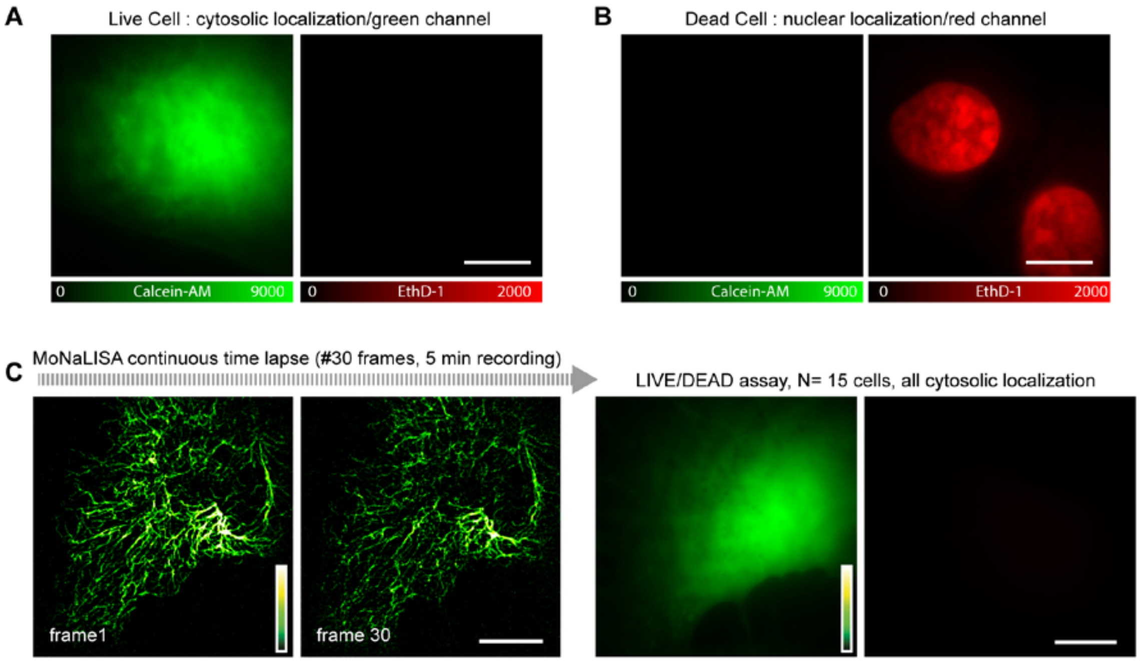

Supplementary Figure 11. LIVE/DEAD assay for cell viability during MoNaLISA imaging. (**A-B**) Control experiments showing the fluorescent signal of the Calcein-AM dye inside a living U2OS cell endogenously expressing vimentin-rsEGFP2 (**A**) and the ethidium homodimer nuclear staining in a dead cell, previously treated with MetOH (**B**). (**C**) Example of a vimentin-rsEGFP2 U2OS cell imaged for 30 frames with MoNaLISA (only the first and the last frames are shown) and then stained with the LIVE/DEAD solutions. The LIVE/DEAD staining is comparable to the one of the control experiment in (**A**): cytosolic green fluorescence and no red nuclear staining. The same experiment has been performed for N=15 cells. All of the cells showed the same positive behavior. Scale bars, 10 µm.

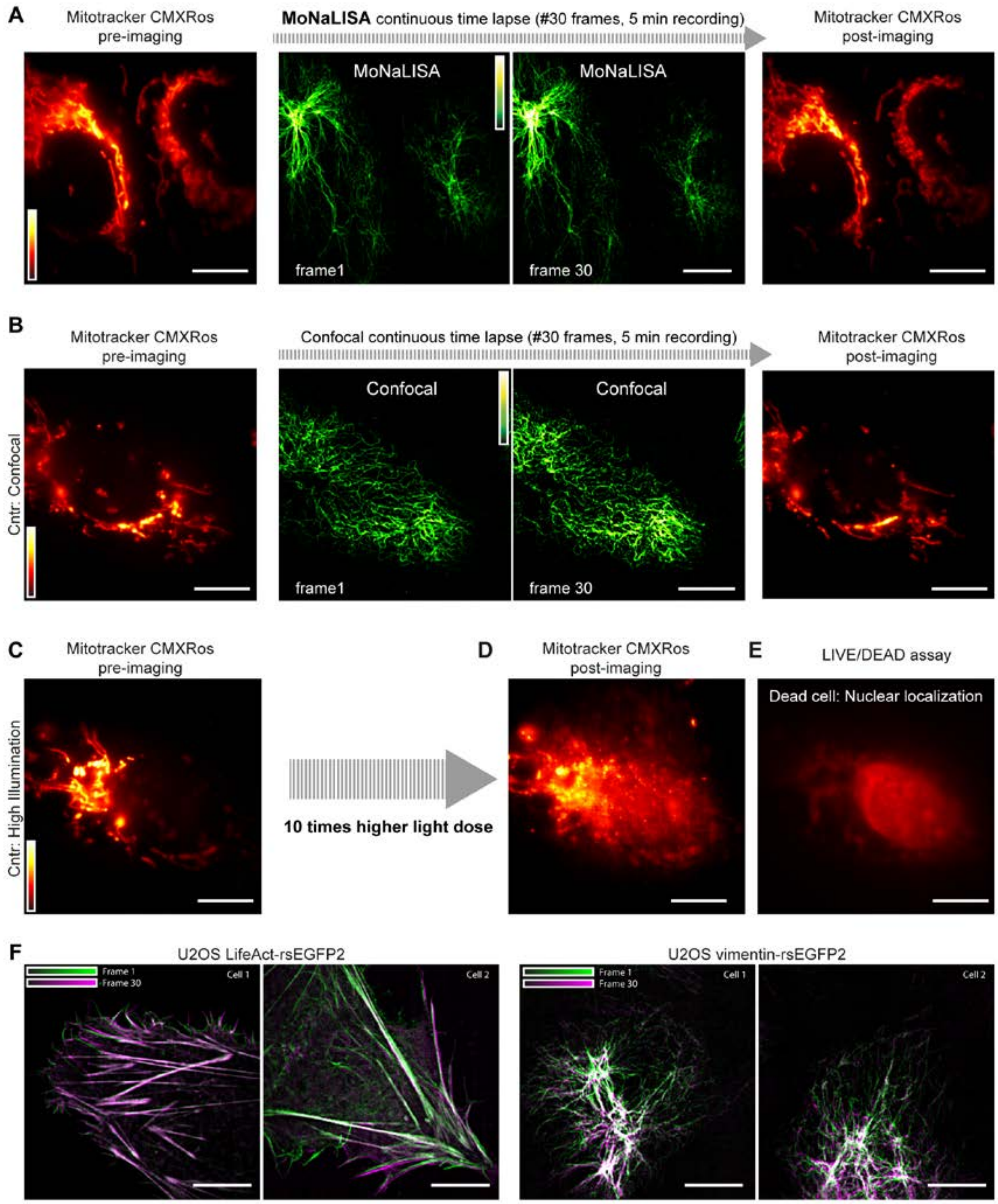

Supplementary Figure 12. Mitochondria morphology and cytoskeleton overall shape during MoNaLISA imaging. (A) Example of a living U2OS cell endogenously expressing vimentin-rsEGFP2 stained with the mitochondrial marker MitoTracker CMXRos and then imaged for 30 frames (only the first and the last frames are shown) with MoNaLISA. The images of mitochondria before and after the time lapse do not show any particular change

in morphology. (**B**) The same experiment has been performed in a confocal mode, as a control, and gave the same results as A. (**C**) The use of a 10 times higher light dose than the one used in MoNaLISA caused the fragmentation/disruption of the mitochondria (**D**). The LIVE/DEAD staining (**E**) on this cell showed a red nuclear staining and no green signal was detected, indicating the death of the cell. (**F**) Monitoring the cytoskeleton through the actin binding protein LifeAct (first two images) and vimentin (last two images) during MoNaLISA recording showed no significant retraction or shrinking of the cell.

Supplementary Table 1. Imaging parameters for the reported experiments.

Fig.	Cell Type	Protein of Interest	rsFP	ON-switching			OFF-switching			Read-Out		
				I (W/cm^2)	time (ms)	J /cm^2	I (W/cm^2)	time (ms)	J /cm^2	I (kW/cm^2)	time (ms)	J /cm^2
2	U2OS	Vim (endogenous)	rsEGFP2	540	0.5	0.27	357	1.5	0.54	0.800	1.5	1.2
3.a	neuron	LifeAct	dronpa(M159T)	540	0.5	0.27	722.5	1	0.72	1	1.5	1.5
3.b	neuron	LifeAct/actinChromobody	rsEGFP2	260	0,5	0,13	360	1.5	0.54	1	1	1
3.c	brainSlice	actinChromobody	rsEGFP2	400	0.5	0.20	365.5	1	0.37	5	1.5	7.5
3.d	ESC	LifeAct	rsEGFP2	140	0.3	0.04	289	1	0.29	1	1.5	1.5
3.e	ESC	LifeAct	rsEGFP2	400	0.5	0.20	361.25	1	0.36	1	1.5	1.5
4.a-b	U2OS	LifeAct	rsEGFP2	260	0.5	0.13	289	1.5	0.43	1	1.5	1.5
4.c-d	neuron	LifeAct	dronpa(M159T)	540	0.5	0.27	748	1	0.75	1	1	1
4.e	neuron	LifeAct	rsEGFP2	260	0,5	0,13	360	1.5	0.54	1	1	1
4.f	U2OS	OMP25	rsEGFP2	830	0.25	0.21	578	0.6	0.35	2.5	0.75	1.9
S5.E	neuron	LifeAct	dronpa(M159T)	540	0.5	0.27	722.5	1	0.72	1	1.5	1.5
S9.A	U2OS	Vim (endogenous)	rsEGFP2	525	0.5	0.26	357	1.5	0.54	1	2	2
S9.B	U2OS	Vim (endogenous)	rsEGFP2	540	0.5	0.27	357	1.5	0.54	1	1.5	1.5
S9.C	U2OS	Vim (endogenous)	rsEGFP2	525	0.5	0.26	544	1.5	0.82	1	1	2
S11.A	astrocite	LifeAct	rsEGFP2	500	0.5	0.25	365.5	1.5	0.55	1	1.5	1.5
S.12-S.13	U2OS	Vim (endogenous)	rsEGFP2	260	0,5	0,13	330	1.5	0.50	1	1	1
M.1	U2OS	LifAact	rsEGFP2	140	0.5	0.07	748	1.5	1.10	1	1.5	1.5
M.4	U2OS	Vim (endogenous)	rsEGFP2	110	0.3	0.03	289	1	0.29	1	1.5	1.5

Fig.	Scan Step x-y / P_{MF} (nm)	Scan Step z (nm) x Number of frames	FOV (µm)	Time Points	dwell time / frame time
2	25/750	-	46 x 46	-	8 ms / 7.2sec
3.a	25/750	100 x 28	46 x 46	-	7.5 ms / 6.8 sec
3.b	35/750	200 x 11	42x42	-	6,5ms /3.1 sec
3.c	35/750	500 x 26	39 x 39	-	6.5 ms / 3.1 sec
3.d	35/750	500 x 22	46 x 46	-	7.5 ms / 3.6 sec
3.e	35/750	250 x 33	39 x 39	2	6.0 ms / 2.9 sec
4.a-b	35/750	-	45.5 x 45.5	45	8 ms / 3.9 sec
4.c-d	35/750	-	45.5 x 45.5	19	7 ms / 3.4 sec
4.e	35/750	-	42x42	10	6,5ms /3.1 sec
4.f	40/750	200 x 4	8 x 39	15	2.3 ms / 0.83 sec
S5.E	35/750	100 x 37	46 x 46	-	7.5 ms / 3.6 sec
S9.A	20/500	-	32 x 32	-	8.5 ms / 5.3 sec
S9.B	30/750	-	48 x 48	-	8 ms / 5 sec
S9.C	20/1250	-	29 x 29	-	7.5 ms / 28 sec
S11.B	25/750	150 x 55	39 x 39	-	6.5 ms / 5.85 sec
S.13-S14	40/750	-	42x42	30	6.5ms /2.3 sec
M.1	40/750	250 x 25	46 x 46	-	8.0 ms / 2.9 sec
M.4	35/750	-	47 x 47	50	7.3 ms / 3.5 sec

Supplementary References

1. A. Chmyrov, J. Keller, T. Grotjohann, M. Ratz, E. d'Este, S. Jakobs, C. Eggeling, S. W. Hell, Nanoscopy with more than 100,000 'doughnuts'. *Nature methods* **10**, 737-740 (2013); published online EpubAug (10.1038/nmeth.2556).
2. Lu Wei, Zhixing Chen, Lixue Shi, Rong Long, Andrew V. Anzalone, Luyuan Zhang, Fanghao Hu, Rafael Yuste, Virginia W. Cornish & Wei Min , Super-multiplex vibrational imaging. *Nature* **544**, 465-470 (2017)
3. Jianjun He, Yi Wang, Maria A Missinato, Ezenwa Onuoha, Lydia A Perkins, Simon C Watkins, Claudette M St Croix, Michael Tsang & Marcel P Bruchez, A genetically targetable near-infrared photosensitizer, *Nature Methods* **13**, 263-268 (2016).
4. Hidenori Otera and Katsuyoshi Mihara, Mitochondrial Dynamics: Functional Link with Apoptosis, *International Journal of Cell Biology*, 821676 (2012)
5. M Karbowski and R J Youle, Dynamics of mitochondrial morphology in healthy cells and during apoptosis, *Cell Death and Differentiation* **10**, 870-880 (2003)
6. Waldchen, S., Lehmann, J., Klein, T., van de Linde, S. & Sauer, M. Light-induced cell damage in live-cell super-resolution microscopy. *Scientific reports* **5**, 15348 (2015).
